# Jealousy in interracial and same-race relationships

**DOI:** 10.1177/02654075251317425

**Published:** 2025-02-01

**Authors:** Vikki Pham, Eri Sasaki, Hanieh Naeimi, Emily A Impett

**Affiliations:** 1 7938 University of Toronto, Canada; 2 7938 University of Toronto 71637 Mississauga, Canada

**Keywords:** Interracial relationships, jealousy, attachment anxiety, couple identity

## Abstract

Interracial relationships have been on the rise and face unique relational challenges but are underrepresented in relationship science which has relied heavily on studies of same-race White couples. Existing research has shown that individuals in interracial relationships experience greater jealousy than those in same-race relationships, but these studies were underpowered or relied on binary measures of jealousy. In a large sample of individuals in interracial (*N* = 196) and same-race relationships (*N* = 198) from the United States and Canada, we found that individuals in interracial relationships reported experiencing jealousy more frequently and intensely (general jealousy), had greater worries about potential romantic rivals (rival-directed cognitive jealousy), and felt more distrust and anger toward rivals (rival-directed emotional jealousy). However, there were no differences in the extent to which they derogated the rival and displayed their relationship in front of the rival (rival-directed behavioral jealousy), and the findings for general and cognitive jealousy became nonsignificant when controlling for attachment anxiety. Finally, having a stronger couple identity attenuated the negative effects of having higher general jealousy and cognitive jealousy on relationship satisfaction for individuals in interracial (but not same-race) relationships. Future research should explore the development of attachment anxiety in interracial relationships and explore strategies in addition to having a stronger couple identity that can help interracial couples navigate third-party threats more effectively.

Interracial romantic relationships, in which partners identify as having different racial backgrounds, have been historically stigmatized and criminalized in some countries including the United States (U.S.; Pascoe, 1991). Laws that prohibited interracial marriages were prevalent in the U.S. as early as 1664. Although these laws were overturned following Loving v. Virginia in 1967, Alabama was the last state to officially remove this ban in 2000, with only 59% support. These marriages were never illegal in Canada, but interracial couples were subjected to hate crimes and discriminated against (Bielski & Chambers, 2017). In recent years, interracial relationships have been on the rise in the U.S. and Canada (U.S. Census Bureau, 2018; Vanier Institute of the Family, 2021) yet relationship science has mostly focused on same-race relationships (Williamson et al., 2022). It is important to study interracial couples because they face unique structural and social challenges, such as discrimination and social disapproval (Bell & Hastings, 2015), that have implications for their relational well-being (Lehmiller & Agnew, 2006).

Given this historical backdrop, individuals in interracial relationships may feel more anxious about their relationship and more threatened by potential romantic rivals (i.e., experience jealousy). Past research comparing jealousy in interracial and same-race relationships has found that individuals in interracial relationships report higher levels of jealousy (Brownridge et al., 2021; Henderson, 2000). Yet, these studies are limited by the use of small samples or binary measures of jealousy. In the present research, our first aim was to test whether individuals in interracial relationships experience greater jealousy than those in same-race relationships using a large sample of participants from the U.S. and Canada. We sought to extend past research by examining specific components of jealousy (cognitive, emotional, and behavioral jealousy). Our second aim was to examine whether the potential negative effects of jealousy on relationship satisfaction can be buffered by having a strong couple identity (i.e., a strong sense of unity between partners).

## The sociopolitical context of interracial relationships

Interracial relationships have historically been viewed as threatening to the racial hierarchies of Western societies because they challenged the existing dominant power structures (Tabili, 2005). This racial landscape gave rise to anti-miscegenation laws in the U.S. for over three centuries, along with negative societal attitudes and public scrutiny across the U.S. and Canada (Thompson, 2009). Although increased globalization and acceptance have contributed to increased rates of interracial relationships, interracial couples continue to face delegitimization (Bell & Hastings, 2015). For example, interracial couples are subject to microaggressions (i.e., subtle discrimination) that question the validity of their relationship. These can include others mistaking partners in interracial relationships for friends, asking the couple invasive questions, or assuming that the relationship is a phase (van der Walt & Basson, 2015). Interracial couples often face disapproval from their families, which challenges their relationship’s legitimacy and imposes emotional stress on the couple (Bell & Hastings, 2015). These experiences of societal rejection may portray interracial relationships as inherently problematic and incompatible with the norms of society.

Experiences of delegitimization may be particularly salient in Black-White interracial couples who face significant discrimination given the history of slavery and social segregation in the U.S. and Canada (Thompson, 2009). Specifically, Black men who are in relationships with White women often face disapproval from the Black community for abandoning their roots (Craig-Henderson, 2006), whereas White women in these relationships are perceived as lower status by the White community (Stillwell & Lowery, 2021). Black women in relationships with White men also report White women disrespecting their relationship by openly flirting with their partner (Gonlin, 2023). While fewer studies have explored the challenges faced by other racial groups within interracial relationships, individuals across various interracial pairings are likely to be hypervigilant in public (Johnson, 2024). This hypervigilance may extend to romantic rivals who pose a threat to their relationship, which may in turn elicit stronger jealousy reactions among individuals in interracial relationships than those in same-race relationships.

## Interracial relationships and jealousy

Only two studies that we know of have examined whether the experiences of jealousy differ in interracial and same-race relationships. In the first study, Henderson (2000) found that interracial couples were more likely than same-race couples to face disagreements related to jealousy, and these discussions were more focused on distrust toward romantic rivals than their partner. Henderson (2000) speculated that because interracial couples face greater discrimination, they may be more vigilant to how others behave toward them (e.g., others acting friendly with their partner). However, Henderson’s findings were based on observations of a small sample of couples in the U.S., with the average age being in their mid-20s. The data for this study was also collected over three decades ago and may not reflect current societal attitudes and experiences. More recently, Brownridge et al. (2021) found in a large sample of Canadians that individuals in interracial relationships were almost twice as likely (5.3%) as those in same-race relationships (2.8%) to perceive their partner as jealous. However, Brownridge and colleagues (2021) focused on perceptions of partner jealousy rather than individuals’ own jealousy and were not able to capture the degree of jealousy experienced since they used a binary response scale (i.e., yes/no) to assess jealousy.

In the present study, our first aim was to replicate and extend prior work to test whether individuals in interracial relationships experience greater jealousy than those in same-race relationships. We used a continuous measure to assess the degree of jealousy experienced in a high-powered sample. First, we assessed *general jealousy*, including the extent to which jealousy is a problem in the relationship and the overall frequency and intensity of jealousy (White, 1981). Second, we examined specific components of jealousy: *cognitive, emotional, and behavioral jealousy*. All three jealousy components can be directed at the partner and/or the romantic rival who threatens one’s relationship. Given the sociopolitical context and social disapproval faced by interracial couples, they may be more wary of others—including potential rivals—disrespecting and threatening their relationship (Henderson, 2000). Thus, we focused specifically on rival-directed jealousy.

Cognitive, emotional, and behavioral jealousy are reflected in the Dynamic Functional Model of Jealousy (Chung & Harris, 2018). This model conceptualizes jealousy as an ongoing process with early and late phases. During the early phase, individuals appraise whether a potential rival is a threat to their relationship. For example, individuals may reflect on whether their partner’s interactions with another person are just them being friendly or indicative of more serious acts of infidelity. *Cognitive jealousy* reflects this early-phase threat appraisal process. Rival-directed cognitive jealousy captures how often individuals have suspicions about potential rivals being interested in their partner.

After perceiving a rival as a threat, the late phase kicks in, whereby individuals experience jealousy-related emotions and employ behaviors aimed at protecting the relationship. *Emotional and behavioral* jealousy reflect these late-phase jealousy responses. Rival-directed emotional and behavioral jealousy includes how much individuals feel anger and distrust toward the rival and how much they derogate the rival. Given the sociopolitical histories and negative societal attitudes, individuals in interracial relationships may feel more anxious about their relationship and more threatened by rivals. Accordingly, they may experience greater general jealousy and rival-directed cognitive, emotional, and behavioral jealousy than those in same-race relationships.

## The role of couple identity

Given past research showing that jealousy undermines relationship satisfaction (Elphinston et al., 2013), our second aim was to identify a protective factor that may reduce the potential negative effects of jealousy. We proposed that a strong *couple identity*—in which partners perceive themselves as a unit—may buffer the negative effects of jealousy on relationship satisfaction. Our assessment of couple identity includes individuals’ sense of “we-ness” with their partner across cognitive (e.g., sharing similar values and goals), emotional (e.g., feeling in sync), and behavioral facets (e.g., sharing quality time; Topcu-Uzer et al., 2021), as well as a sense of being interconnected with their partner (Aron et al., 1992). Couple identity can serve as a resource that helps couples become more resilient in the face of challenges. For example, intercultural couples with a strong couple identity are better able to navigate their cultural differences and capitalize on opportunities for growth (West et al., 2024). A strong couple identity also improves individual and relational adjustment in the face of chronic diseases (Helgeson et al., 2017; Huang et al., 2018). Similarly, when facing the threat of a romantic rival, a strong couple identity may fortify the couple’s union and protect their relationship from the negative effects of jealousy.

Rather than focusing on one’s own anxieties or placing blame on a partner, couples with a strong couple identity may be more likely to view the threat of a romantic rival as a shared problem and work together as a team (Helgeson et al., 2017). A strong couple identity also helps couples construct meaning from the third-party incident which can provide direction for moving forward (Skerrett, 2015). At the same time, a strong couple identity fosters a sense of safety within the relationship, providing a foundation for couples to support each other as they address challenges together (Reid & Ahmad, 2015). Thus, a strong couple identity may help couples better navigate the threat of a romantic rival, thereby protecting individuals who feel jealous from experiencing lower relationship satisfaction.

## Overview of the current study

We conducted a cross-sectional study of individuals involved in interracial and same-race romantic relationships to test two key aims. First, we compared the different components of jealousy among individuals in interracial and same-race relationships (Aim 1). We hypothesized that individuals in interracial relationships, compared to those in same-race relationships, will report greater general jealousy and rival-directed cognitive, emotional, and behavioral jealousy. Second, we examined whether having a strong couple identity buffered any negative effects of jealousy on relationship satisfaction (Aim 2). We hypothesized that the negative effect of jealousy on relationship satisfaction will be attenuated for individuals who have a stronger (vs. weaker) couple identity. We pre-registered our hypotheses and analyses (https://osf.io/ck2sm/).^1^

We made three key deviations from our pre-registered analytic plan. First, we examined whether there were differences between individuals in interracial and same-race relationships in their demographics (i.e., age, relationship length) and relationship satisfaction. If so, in subsequent analyses, we controlled for these variables to examine the robustness of the effects. Second, given previous research linking attachment insecurity with jealousy (Huelsnitz et al., 2018; Sharpsteen & Kirkpatrick, 1997), we ran additional analyses controlling for attachment anxiety and avoidance to determine if the effects remain. Third, in examining Aim 2, we ran additional analyses modeling possible three-way interactions between the type of relationship, couple identity, and jealousy predicting relationship satisfaction. This allowed us to examine whether the hypothesized buffering effects of couple identity might differ for individuals in interracial versus same-race relationships.

## Method

### Participants

A priori power analyses indicated a minimum sample size of 396 participants (see Online Supplemental Materials [OSM] at https://osf.io/ck2sm/). To account for exclusions, we oversampled and recruited 500 participants involved in romantic relationships (240 interracial, 260 same-race) in the U.S. and Canada via CloudResearch, an online research platform that yields representative samples and high-quality data (Hauser et al., 2022). Unknown to participants, the survey was made available only to those in interracial or same-race relationships through the use of demographic screeners on CloudResearch (i.e., participants did not know that they were being recruited because they were in a particular type of relationship). We excluded participants who failed attention checks (*n* = 54), admitted to responding dishonestly (*n* = 2), or requested to withdraw their data (*n* = 31). We also excluded participants whose responses on the CloudResearch screener regarding whether they were in an interracial or same-race relationship were inconsistent with their reports of their own and their partner’s race in our survey (*n* = 14; see OSM Table S1). Our analyses were based on a final sample of 394 participants (196 in interracial relationships, 198 in same-race relationships).

The sample comprised 51% of participants identifying as cisgender men, 48% as cisgender women, 0.8% as non-binary, and 0.3% as transgender men. Participants’ average age was 38.81 years (*Mdn* = 37.00, *SD* = 10.23; range = 19–73), and their average relationship length was 10.37 years (*Mdn* = 7.25, *SD* = 9.25; range = 3 months to 59.92 years). Most participants were married (51.5%) or engaged (7.4%), and the remaining were in common-law relationships (6.6%) or dating (34.5%). Most participants were cohabiting with their partners (86%). Most participants identified as heterosexual (83.2%), 7.9% identified as bisexual, 5% identified as gay or lesbian, 1.3% identified as pansexual, 1% identified as queer, 1% identified as asexual, and 0.5% identified as having another sexual orientation. Most participants were employed full-time (76.7%), while 14.5% were employed part-time, 8.4% were unemployed, and 0.5% were students. On a 10-rung ladder where the top rung represents people with the highest education, income, and occupation status (MacArthur Scale of Subjective Social Status; Adler et al., 2000), participants’ average placement on the ladder was at the fifth rung (*Mdn* = 5.00, *SD* = 1.62; range = 1–10). While the sample of participants in *same-race* relationships was mostly White (78.3%; 10.1% Black, 6.6% Asian, 4.5% Latin American, 0.5% Native American), the sample of participants in *interracial* relationships was more racially diverse (49% White, 15.8% Black, 10.2% Asian, 13.8% Latin American, 1% Native American, and 10.2% Bi/Multiracial). The sample of participants’ partners in interracial relationships was also racially diverse (34.2% White, 13.3% Black, 24.5% Asian, 16.8% Latin American, 10.2% Bi/Multiracial, and 1% Pacific Islander). However, most of the racial combinations of participants and their partners in interracial relationships involve one White partner (see Table 1).

**Table 1.** Frequency of racial combinations in interracial relationships.

Racial combination (participant-partner)	% interracial relationships
White-Black	10.7
White-Asian	17.86
White-Latin American	13.27
White-Bi/Multiracial	6.6
White-Pacific Islander	0.5
Black-White	8.2
Black-Asian	3.1
Black-Latin American	3.1
Black-Bi/Multiracial	1.5
Asian-White	9.2
Asian-Latin American	0.5
Asian-Bi/Multiracial	0.5
Latin American-White	9.7
Latin American-Black	1.0
Latin American-Asian	2.6
Latin American-Pacific Islander	0.5
Native American-White	1.0
Bi/Multiracial-White	6.6
Bi/Multiracial-Black	1.5
Bi/Multiracial-Asian	1.5
Bi/Multiracial-Bi/Multiracial	0.5

### Measures and procedure

After providing informed consent, participants completed an online survey. At the end of the survey, participants read a debriefing statement and were compensated 4.80 USD. For each measure, the items were averaged (see Table 2). Table 3 presents correlations among all measures.

**Table 2.** Descriptive statistics of all measures.

			Range		
Measures	*M*	*SD*	Lower	Upper	α
General Jealousy	2.93	1.74	1.00	7.00	.94
Rival-Directed Jealousy					
Cognitive Jealousy	2.98	1.74	1.00	7.00	.96
Emotional Jealousy	5.54	1.62	1.00	7.00	-
Behavioral Jealousy	3.16	1.76	1.00	7.00	.93
Couple Identity					
We-ness	4.22	0.75	1.00	5.00	.96
Inclusion of Other in the Self	5.62	1.56	1.00	7.00	-
Relationship Satisfaction	5.53	1.37	1.00	7.00	.95
Attachment Insecurity					
Attachment Anxiety	3.06	1.20	1.00	6.33	.84
Attachment Avoidance	3.54	1.31	1.00	6.75	.88
Social Disapproval	1.93	1.10	1.00	6.17	.85

*Note.* The We-ness Questionnaire represents averages of items on 5-point scales. All other measures represent averages of items on 7-point scales.

**Table 3.** Correlations among study variables.

Variables	1	2	3	4	5	6	7	8	9
1. General Jealousy	-								
2. Cognitive Jealousy	.820***	-							
3. Emotional Jealousy	.221***	.300***	-						
4. Behavioral Jealousy	.553***	.495***	.426***	-					
5. Couple Identity	-.185***	-.157**	.153**	.034	-				
6. Relationship Satisfaction	-240***	-.212***	.115*	-.022	.832***	-			
7. Attachment Anxiety	.498***	.441***	.019	.257***	-.383***	-.373***	-		
8. Attachment Avoidance	.230***	.203***	-.018	.138**	-.255***	-.254***	.424***	-	
9. Social Disapproval	.343***	.330***	-.014	.122*	-.534***	-.515***	.466***	.237***	-

*Note.* Bolded correlations are significant. *p < .05, **p < .01, ***p < .001.

#### General Jealousy

To assess general jealousy, participants rated four items from a modified version of White’s (1981) Relationship Jealousy Scale (e.g., “How often do you get jealous in your current relationship?“). These items were rated on a 7-point scale with different anchors (e.g., 1 = *not at all*, 7 = *very much*).

### Rival-directed jealousy

#### Cognitive Jealousy

Participants rated five items adapted from the Cognitive Subscale of the Multidimensional Jealousy Scale (Pfeiffer & Wong, 1989). These items were modified to capture rival-directed cognitive jealousy by focusing on suspicious worries about others being attracted to a partner as opposed to one’s own partner being attracted to others (e.g., “I am worried that someone else is trying to seduce my partner”; 1 = *never*, 7 = *all the time*). These items showed high reliability. Given that our conceptualization of cognitive jealousy reflects the early phase of assessing whether a potential rival is a threat to the relationship before the threat is realized (Chung & Harris, 2018), we measured cognitive jealousy before asking participants to recall an instance of a third-party threat.

#### Jealousy Recall

Emotional and behavioral jealousy reflect the late-phase manifestations once a potential rival is perceived as a threat, and thus were measured after asking participants to recall an instance of a third-party threat using the following instructions (adapted from Guerrero et al., 2011):

Jealousy is a common human emotion. In fact, most people experience jealousy at some point in their romantic relationships. Jealousy occurs when a person believes that a third party (sometimes called a “rival”) threatens their romantic relationship in some way. For example, a person may feel jealous when someone else flirts with their partner.

Please think about a time when you believed that a third party has threatened your current romantic relationship in some way. Please describe this episode, including what happened, and how you felt about it.

This jealousy recall task allowed participants to relive the experience of facing a third-party threat so that they could answer the subsequent questions about their emotional and behavioral jealousy reactions. Some common jealousy-eliciting events that participants described included an attractive third party (e.g., coworker, friend, neighbor, ex-partner) being overly friendly toward, spending a lot of time with, or appearing to have feelings for their partner. Responses related to race or culture were less common, with only four participants in interracial relationships mentioning someone speaking to their partner in their partner’s native language, which participants could not understand. Participants who did not provide valid responses to this question (e.g., had not experienced jealousy, described a hypothetical scenario, or otherwise failed to answer the question; *n* = 34) were excluded from the analyses that modeled emotional and behavioral jealousy.

#### Emotional Jealousy

Participants rated the extent to which they felt six emotions when a third party had threatened their current romantic relationship: “jealous”, “betrayed”, “distrustful”, “angry”, “worried”, and “hurt” (1 = *not at all*, 7 = *very much*; Parrott & Smith, 1993). To assess rival-directed emotional jealousy, participants who responded with more than a “1” to feeling “distrustful” and “angry” rated how strongly they felt these emotions towards the rival (*r* = .74).

#### Behavioral Jealousy

Participants rated five items from the Communicative Responses to Jealousy Scale (Guerrero et al., 2011) regarding how much they derogated the rival (e.g., “I pointed out the rival’s bad qualities”) along with three items on how much they displayed their relationship in front of the rival (e.g., “I showed my partner extra affection when rivals were around”; 1 = *never*, 7 = *always*).

#### Couple Identity

Participants completed the 17-item We-ness Questionnaire (e.g., “We share similar meanings about life”; 1 = *not at all*, 5 = *very much*; Topcu-Uzer et al., 2020) as well as the one-item Inclusion of the Other in the Self scale (IOS; Aron et al., 1992). The IOS scale comprised seven pairs of circles representing the self and the partner, overlapping to varying degrees (1 = *circles not overlapping at all,* 7 = *circles overlapping completely*). Participants selected the pair of circles that best described their relationship with their partner. The scores on the We-ness questionnaire and IOS Scale were highly correlated (*r* = .66) and were standardized and combined for analyses.

#### Relationship Satisfaction

To assess relationship satisfaction, participants completed the five-item satisfaction subscale of the Investment Model Scale (Rusbult et al., 1998; e.g., “I feel satisfied with our relationship”; 1 = *strongly disagree*, 7 = *strongly agree*).

#### Attachment Insecurity

Participants completed the 17-item Adult Attachment Questionnaire (Simpson et al., 1996) to assess attachment anxiety (e.g., “I often worry that my partner(s) don’t really love me”) and attachment avoidance (e.g., “I find it difficult to trust others completely”; 1 = *strongly disagree*, 7 = *strongly agree*).

## Results

### Aim 1: Interracial versus same-race relationships and Jealousy

We conducted a series of one-way ANOVAs to test our first hypothesis that individuals in interracial relationships would experience greater levels of jealousy than those in same-race relationships. Replicating previous research, individuals in interracial relationships (*M* = 3.15, *SD* = 1.82) reported greater *general jealousy* (i.e., more frequent and intense feelings of jealousy) than those in same-race relationships (*M* = 2.71, *SD* = 1.62), *F*(1, 392) = 6.26, *p* = .013, η_p_^2^ = .07.

We found partial support for the rival-directed components of jealousy. First, individuals in interracial relationships (*M* = 3.22, *SD* = 1.87) reported greater *cognitive jealousy* (i.e., suspicious worries about potential rivals) than those in same-race relationships (*M* = 2.74, *SD* = 1.56), *F*(1, 392) = 7.77, *p* = .006, η_p_^2^ = .00. Second, individuals in interracial relationships (M = 5.75, SD = 1.56) reported greater emotional jealousy (i.e., distrust and anger toward the rival) than those in same-race relationships (M = 5.40, SD = 1.64), F(1, 327) = 3.84, *p* = .051, ηp^2^ = .34. Finally, there was no significant difference between individuals in interracial (*M* = 3.31, *SD* = 1.81) and same-race (*M* = 3.14, *SD* = 1.67) relationships in their levels of *behavioral jealousy* (i.e., derogating the rival and displaying their relationship in front of the rival), *F*(1, 359) = 0.80, *p* = .372, η_p_^2 ^< .08.^2,^^3^

We then conducted additional analyses to control for any differences between participants in interracial versus same-race relationships. An independent samples *t*-test revealed that participants in interracial (*M* = 5.49, *SD* = 1.40) and same-race relationships (*M* = 5.56, *SD* = 1.34) did not differ in their relationship satisfaction, *t*(392) = -0.54, *p* = .593, *d* = .05. However, participants in interracial relationships were younger (*M* = 37.21 years, *Mdn* = 36.00, *SD* = 8.91) than participants in same-race relationships (*M* = 40.37 years, *Mdn* = 38.00, *SD* = 11.17), *t*(373) = -3.09, *p =* .002, *d* = .31. Participants in interracial relationships also had shorter relationships (*M* = 9.19 years, *Mdn* = 6.17, *SD* = 8.68) than those in same-race relationships (*M* = 11.54 years, *Mdn* = 8.13, *SD* = 9.66), *t*(388) = -2.54, *p* = .012, *d* = .26. Nevertheless, rerunning our analyses testing the significant effects of relationship type (interracial = 1 and same-race = 0) on jealousy (general jealousy and rival-directed cognitive and emotional jealousy) controlling for the effects of age and relationship length yielded similar results (see OSM Table S6).

We also conducted additional analyses to test whether the significant effects of relationship type on general jealousy and rival-directed cognitive and emotional jealousy were independent of attachment insecurity. We conducted regression analyses to model the degree to which relationship type, attachment anxiety, and attachment avoidance predicted each jealousy outcome that was significant in previous analyses. As shown in Table 4, the effects of interracial versus same-race relationships on *general jealousy* and rival-directed *cognitive jealousy* became nonsignificant, whereas the effects of attachment anxiety were significant, with higher attachment anxiety predicting greater general and cognitive jealousy. These findings suggest that the effects of relationship type on general jealousy and cognitive jealousy might have emerged because individuals in interracial relationships tended to be higher in attachment anxiety. With regard to rival-directed *emotional jealousy*, the effect of relationship type remained significant, whereas the effects of attachment anxiety and attachment avoidance were nonsignificant. Thus, the effect on distrust and anger towards the rival was unique to whether individuals were in interracial versus same-race relationships.

**Table 4.** The effects of relationship type, attachment anxiety, and attachment avoidance on jealousy.

			95% CI			
Predictors	*B*	*t*	Low	High	*p*	*r*
Effects on General Jealousy						
Intercept	2.84	26.32	2.628	3.053	< .001	.80
Relationship Type	0.18	1.16	-0.124	0.482	.247	.06
Attachment Anxiety	**0.70**	**9.91**	**0.560**	**0.837**	**< .001**	**.45**
Attachment Avoidance	0.03	0.39	-0.101	0.151	.697	.02
Effects on Cognitive Jealousy						
Intercept	2.85	25.53	2.627	3.066	< .001	.79
Relationship Type	0.26	1.63	-0.054	0.573	.104	.08
Attachment Anxiety	**0.62**	**8.45**	**0.472**	**0.758**	**< .001**	**.39**
Attachment Avoidance	0.02	0.26	-0.113	0.147	.797	.01
Effects on Emotional Jealousy						
Intercept	5.41	43.01	5.158	5.652	< .001	.92
Relationship Type	**0.35**	**1.95**	-**0.003**	**0.701**	**.052**	**.11**
Attachment Anxiety	0.01	0.12	-0.152	0.172	.905	.01
Attachment Avoidance	-0.03	-0.39	-0.181	0.121	.696	.03

*Note.* Relationship type was dummy coded (0 = same-race relationship and 1 = interracial relationship). CI = confidence interval. *Df* = 390 for the models predicting general jealousy and cognitive jealousy; *df* = 325 for the models predicting emotional jealousy. Effect sizes (*r*) were computed using Rosnow and Rosenthal’s (2007) formula: *r* = √(*t^2^* / *t^2^* + *df*).

The finding that individuals in interracial relationships were higher in attachment anxiety than those in same-race relationships prompted us to conduct additional analyses to identify a potential explanation for this effect. We reasoned that greater social disapproval, commonly experienced by individuals in interracial relationships (Rosenthal & Starks, 2015), might heighten their fears of rejection and account for their reportedly higher attachment anxiety compared to those in same-race relationships. We did include the 6-item Relationship Stigma Scale (Rosenthal & Starks, 2015) to assess social disapproval. In a set of additional non-preregistered exploratory analyses, we found that individuals in interracial (vs. same-race) relationships experienced greater social disapproval, *b* = 0.24, *SE* = .11, *t*(392) = 2.15, *p* = .032, *r* = .11, 95% CI [.021, .455], and social disapproval predicted greater attachment anxiety, *b* = 0.49, *SE* = .05, *t*(391) = 10.17, *p* < .001, *r* = .46, 95% CI [.399, .591]. The indirect effect of relationship type on attachment anxiety through social disapproval was significant (indirect effect = .12, 95% CI [.013, 225]). These results suggest that social disapproval partly explained why individuals in interracial (vs. same-race) relationships were higher in attachment anxiety.

### Aim 2: The role of couple identity

**Figure 1.**
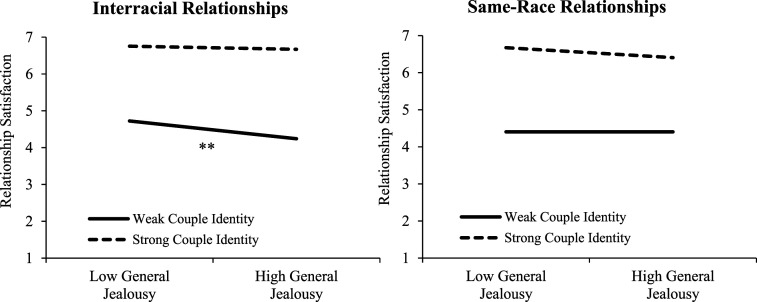
Low and high levels of general jealousy and couple identity represent 1 *SD* below and above the mean. The simple effect of the slope is marked ^**^p < .01. Three-Way Interaction between Relationship Type, General Jealousy, and Couple Identity.

**Figure 2.**
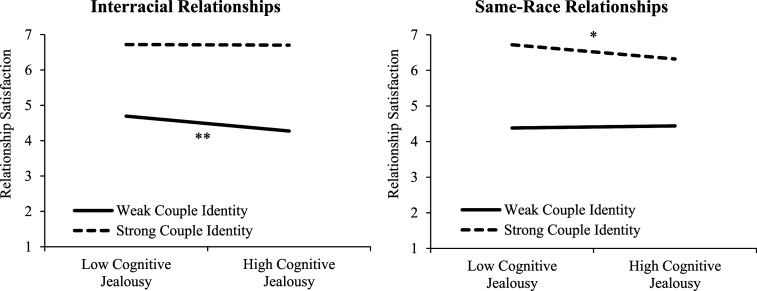
Low and high levels of cognitive jealousy and couple identity represent 1 *SD* below and above the mean. The simple effects of the slopes are marked ^*^p < .05 and ^**^p < .01. Three-Way Interactions between Relationship Type, Cognitive Jealousy, and Couple Identity.

Our second aim was to examine whether having a strong couple identity would attenuate the negative effects of jealousy on relationship satisfaction. To test this hypothesis, we conducted hierarchical regression analyses modeling the degree to which each jealousy component, couple identity, and the interaction between each jealousy component and couple identity predicted relationship satisfaction while controlling for attachment insecurity. As shown in Table 5, no significant interactions between any type of jealousy and couple identity emerged. However, in a set of non-preregistered exploratory analyses, we tested possible three-way interactions between relationship type (interracial = 1 and same-race = 0), each jealousy component, and couple identity on relationship satisfaction while controlling for attachment insecurity. A sensitivity analysis revealed that to achieve 80% statistical power with our current sample size, the smallest effect size we could detect for the three-way interaction was *f*^2^ = .02 (i.e., a small effect; see OSM). We found significant three-way-interactions for both general jealousy (*b* = 0.11, *t* = 2.20, *p =* .028, *r* = .11) and cognitive jealousy (*b* = 0.14, *t* = 2.70, *p =* .007, *r* = .14), but not for emotional jealousy (*b* = 0.05, *t* = 0.94, *p = .*346., *r* = .05) or behavioral jealousy (*b* = 0.05, *t* = 0.87, *p =* .384, *r* = .05). As shown in Figures 1 and 2, decomposing the three-way interactions revealed that for individuals in interracial relationships, those who experienced higher general jealousy and cognitive jealousy reported lower relationship satisfaction when they had a weaker couple identity (*b* = -0.14, *t* = -3.13, *p* = .002, *r* = .16 and *b* = -0.12, *t* = -2.75, *p* = .006, *r* = .14), but having a stronger couple identity buffered the negative effects on relationship satisfaction (*b* = -.02, *t* = -0.57, *p* = .567, *r* = .03 and *b* = -.01, *t* = -.12, *p* = .904, *r* = .01 for general and cognitive jealousy, respectively). In contrast, for individuals in same-race relationships, general jealousy was not associated with relationship satisfaction regardless of having a weaker couple identity (*b* < 0.01, *t* < .01, *p* = .998, *r* < .01) or a stronger couple identity (*b* = -0.08, *t* = -1.70, *p* = .089, *r* = .09). Surprisingly, individuals in same-race relationships who experienced higher cognitive jealousy reported lower relationship satisfaction when they had a stronger couple identity (*b* = -0.12, *t* = -2.36, *p* = .019, *r* = .12) but not a weaker couple identity (*b* = 0.02, *t* = 0.33, *p* = .743, *r* = .02).

**Table 5.** The effects of jealousy and couple identity on relationship satisfaction.

	Relationship satisfaction					
			95% CI			
Predictors	*b*	*t(df)*	Low	High	*p*	*r*
General Jealousy × Couple Identity						
Intercept	5.53	145.85 (389)	5.451	5.600	< .001	.99
General Jealousy	-0.06	-2.44 (389)	-0.111	-0.012	.015	.12
Couple Identity	1.21	26.70 (389)	1.123	1.302	< .001	.80
General Jealousy × Couple Identity	0.02	0.74 (388)	-0.029	0.064	.461	.04
Cognitive Jealousy × Couple Identity						
Intercept	5.53	145.72 (389)	5.451	5.600	< .001	.99
Cognitive Jealousy	-0.06	-2.29 (389)	-0.104	-0.008	.023	.12
Couple Identity	1.20	26.68 (389)	1.124	1.303	< .001	.80
Cognitive Jealousy × Couple Identity	0.01	0.30 (388)	-0.042	0.057	.761	.02
Emotional Jealousy × Couple Identity						
Constant	5.52	134.30 (324)	5.435	5.596	< .001	.99
Emotional Jealousy	-0.01	-0.28 (324)	-0.058	0.044	.783	.02
Couple Identity	1.23	25.20 (324)	1.135	1.328	< .001	.81
Emotional Jealousy × Couple Identity	0.01	0.35 (323)	-0.039	0.056	.725	.02
Behavioral Jealousy × Couple Identity						
Intercept	5.52	143.65 (356)	5.447	5.598	< .001	.99
Behavioral Jealousy	-0.03	-1.36 (356)	-0.077	0.014	.175	.07
Couple Identity	1.23	26.00 (356)	1.137	1.323	< .001	.81
Behavioral Jealousy × Couple Identity	0.01	0.48 (355)	-0.039	0.065	.632	.03

*Note.* We controlled for attachment anxiety and avoidance in these models. CI = confidence interval. Effect sizes (*r*) were computed using Rosnow and Rosenthal’s (2007) formula: *r* = √(*t^2^* / *t^2^* + *df*).

## Discussion

This study contributes to relationship science by studying interracial relationships, a sample that has been underrepresented in the field. We sought to understand jealousy experiences among individuals in interracial and same-race relationships and possible ways to mitigate potential harmful consequences associated with jealousy.

### Comparing jealousy in interracial and same-race relationships

Our first aim was to provide a high-powered test of the link between relationship type (interracial vs. same-race) and jealousy, a finding that had previously been documented in two previous studies that were limited in the use of a small sample (Henderson, 2000) or a binary measure of jealousy (Brownridge et al., 2021). In addition to examining general experiences of jealousy, we drew on the Dynamic Functional Model of Jealousy (Chung & Harris, 2018) to consider the different components of jealousy.

The most robust finding that remained after controlling for attachment insecurity was that individuals in interracial (vs. same-race) relationships felt more distrustful and angrier toward romantic rivals (emotional jealousy). This was consistent with Henderson’s (2000) observations that interracial couples’ discussions of jealousy primarily focused on distrust of rivals as opposed to distrust of their partner. Such distrust toward rivals may stem from their experiences of delegitimization, which may contribute to them feeling disrespected and more wary of how others behave toward their partners. As for general and cognitive jealousy, the effects of relationship type became nonsignificant when controlling for attachment anxiety, whereas the effects of attachment anxiety were significant. These findings suggest that individuals in interracial relationships tend to be higher in attachment anxiety, and attachment anxiety may be driving the effects on general and cognitive jealousy. Moreover, we found that social disapproval may partly explain why individuals in interracial relationships were higher in attachment anxiety, which may have contributed to them experiencing greater general and cognitive jealousy than those in same-race relationships. Interracial couples, who are subjected to societal rejection and delegitimization, may realize that others do not respect their relationship (Gonlin, 2023). This may give rise to the development of attachment anxiety (i.e., chronic fears of rejection) among individuals in interracial relationships, resulting in more frequent and intense experiences of jealousy and increased suspicions about potential rivals.

We did not find that individuals in interracial and same-race relationships differed in their levels of behavioral jealousy. Given that our measure of behavioral jealousy only included items about derogating the rival and displaying their relationship in front of the rival, our scale may be limited in that it did not capture other behavioral expressions of jealousy (Guerrero et al., 2011). It is possible that individuals in interracial relationships may be more inclined to discuss their jealous feelings with their partner, as it may be easier to talk about jealousy arising from distrust toward a third party as opposed to distrust toward their partner. Future research should examine the types of behaviors in which individuals in interracial relationships might engage to protect their relationships.

### The role of couple identity

Our second aim was to investigate whether having a stronger couple identity attenuated the negative effects of jealousy on relationship satisfaction. Although there were no significant two-way interaction effects of couple identity and jealousy, tests of three-way interactions with relationship type revealed that a stronger couple identity buffered the negative effects of general jealousy and rival-directed cognitive jealousy only among those in interracial relationships. These findings are consistent with past research showing the benefits of having a strong couple identity for marginalized couples. For example, in a study on lesbian couples, a sense of couple unification (e.g., “us against the world”) promoted greater recovery from systematic prejudices and adversities, building couple resilience (Connolly, 2005). Similarly, for individuals in interracial relationships, a strong couple identity may strengthen their union against romantic rivals, especially since their jealousy responses may involve distrust of rivals. A strong couple identity in interracial relationships likely fosters mutual trust (Reid & Ahmad, 2015), helping couples work together as a team to overcome frequent or intense jealousy (general jealousy) and worries about potential rivals (cognitive jealousy). Future research should explore other protective factors that can help interracial couples navigate jealousy. For example, perceived partner commitment may signal that a partner is invested in the relationship and will establish clear boundaries with the third-party, which may be particularly beneficial for alleviating feelings of anxiety in interracial couples.

Our results suggest that for individuals in same-race relationships, having a stronger (vs. weaker) couple identity may not provide similar protective effects against general and cognitive jealousy. While we are cautious in interpreting this finding, it aligns with prior research suggesting that the protective effects of couple identity may vary by context (Huang et al., 2018). Future research should consider how couple identity may function differently across different populations in relation to the specific challenge faced, in order to tailor interventions that effectively address the unique experiences of each group.

### Limitations, future directions, and implications

There are several limitations in the present study. First, the correlational nature of the data prevents conclusions about the direction of effects. Although it is possible that more anxiously attached or jealousy-prone individuals might be more likely to enter interracial relationships, we think that our theorized direction of effects is more likely. Specifically, individuals in interracial (vs. same-race) relationships should be more likely to develop higher attachment anxiety and experience greater jealousy due to the structural and social challenges they face, which may contribute to their heightened fears of rejection. Future research could employ experience sampling or longitudinal designs to gain insights into the direction of effects. Future research could also conduct dyadic studies to gain insight into how both partners navigate third-party threats.

Second, we focused on relational characteristics (e.g., interracial vs. same-race relationships, couple identity) and individual differences (e.g., attachment insecurity) influencing the experience of jealousy, but other factors could also play a role. One factor that has received little attention in past research is the rival’s characteristics. For interracial couples, the rival’s race may be especially important. Given the historical context of interracial relationships (Tabili, 2005) and the social disapproval they face (Rosenthal & Starks, 2015), a rival from the same race as one’s partner may be perceived as more threatening. This rival could be perceived as more readily accepted by their partner’s friends and family and may seem to have a mutual understanding of their partner’s racial and cultural background, potentially eliciting stronger jealousy reactions. Moreover, past work on Black-White interracial relationships has found that White women openly disrespect and challenge White men’s commitment to Black women (Gonlin, 2023), and such attempts by same-race rivals to undermine interracial relationships may escalate feelings of jealousy. Future research should explore how the rival’s characteristics influence jealousy outcomes.

Third, we focused on interracial relationships, compared to same-race relationships, to replicate past research on jealousy experiences in these groups. Consistent with previous research (Albuja et al., 2022; Naeimi et al., 2024), we conceptualized race as a social construct that categorizes individuals based on their perceived inherent biological traits, and we defined culture as a system of shared norms, values, and beliefs, passed down within a social group. Race is often considered an indicator of culture, alongside factors like ethnicity or nationality, and is sometimes viewed as a component of culture (Albuja et al., 2022). Thus, our findings may be relevant to intercultural or interfaith relationships, given that these couples also have to navigate differences related to their cultural and religious backgrounds. Like interracial couples, the relational quality of intercultural and interfaith couples is affected by familial and peer disapproval and discrimination from others (Yurtaeva & Charura, 2024). These social and structural challenges may contribute to the development of attachment anxiety and experiences of jealousy in intercultural and interfaith couples, although perhaps to a different extent than in interracial couples.

Fourth, given the interest of time and funding, we focused on residents of the U.S. and Canada. Most of the interracial pairings in our sample involved one White partner, which reflects the U.S. and Canadian context. However, individuals in different interracial pairings may vary in their experiences of jealousy. For example, Black-White interracial couples, who face significant social disapproval from both their own racial community and the general public (Bell & Hastings, 2015), may be more wary of potential romantic rivals and experience heightened jealousy compared to interracial relationships involving other racial groups. Future research should explore whether structural challenges contribute to varying levels of jealousy across different interracial pairings, as well as the extent to which a strong couple identity can buffer these effects.

Our focus on participants from the U.S. and Canada limits generalizability to other sociopolitical and sociocultural contexts that may be more or less accepting of interracial couples. The U.S. and Canada are more racially and culturally diverse compared to many other countries (e.g., Italy, Norway, Japan). Thus, it is possible that interracial couples in these countries may be better accepted than in more homogenous countries, which may influence their levels of jealousy. We did not ask participants to report whether they reside in the U.S. or Canada, so we were not able to examine country-level differences in their experiences of jealousy. Interracial couples in Canada may have different experiences due to country-level policies that promote multiculturalism and integration of different cultures in society, compared to those in the U.S. which has some policies that promote colorblindness. Additionally, although Canada never had specific laws against interracial marriage, individuals in these relationships were still punished and marginalized (Bielski & Chambers, 2017). Yet, without official anti-miscegenation laws, interracial couples in Canada might experience more acceptance than those in the U.S. Future research is encouraged to explore how variations in societal context, such as acceptance of interracial couples, may influence their jealousy experiences.

Fifth, we did not ask participants about their disability status. Having a disability can add to the experience of having a marginalized identity. Individuals with multiple marginalized identities may have different experiences of jealousy due to the unique challenges arising from these intersecting identities. Future research should explore the intersectional effects of multiple marginalized identities on jealousy outcomes.

The findings of our study have implications for couples’ interventions. To help couples more effectively navigate threats related to potential rivals, relationship therapists need to tailor their strategies to suit the unique experiences of interracial and same-race couples. For interracial couples, it may be beneficial to help them navigate experiences of social disapproval to reduce the development of attachment anxiety, which can contribute to jealousy. To alleviate rejection concerns associated with attachment anxiety in interracial relationships, partners can offer reassurance that they are not attracted to potential rivals and reaffirm their love (see Overall et al., 2022). It would also be advantageous for interracial couples to develop a strong sense of couple identity to strengthen their union against third-party threats. In contrast, same-race couples may benefit more from discussions about partner betrayal (Henderson, 2000), but future research is needed to better understand the strategies that can help same-race couples navigate jealousy.

## Conclusion

This study provides the first high-powered examination of jealousy among individuals in interracial and same-race relationships. Compared to those in same-race relationships, individuals in interracial relationships reported experiencing jealousy more frequently and intensely, had greater worries about potential rivals, and felt more distrustful and angrier toward rivals, but were not more likely to derogate the rival or display their relationship in front of the rival. However, the effects for general experiences of jealousy and worries about rivals became nonsignificant when controlling for attachment anxiety. This led to an unanticipated finding that individuals in interracial relationships reported greater attachment anxiety than those in same-race relationships, in part due to greater social disapproval. Finally, having a stronger couple identity attenuated the negative effects of general experiences of jealousy and worries about rivals on relationship satisfaction for individuals in interracial, but not same-race, relationships. Our findings suggest that greater social disapproval faced by interracial couples may contribute to relational challenges, including increased attachment anxiety and jealousy. Future research should explore how the structural and social challenges may contribute to the development of attachment anxiety among interracial couples, as well as other strategies–in addition to having a stronger couple identity–that can help them navigate third-party threats more effectively.

## Notes

1 In our preregistration, we indicated that we would test whether greater vigilance to external threats might explain why individuals in interracial (vs. same-race) relationships experience greater jealousy. However, because of the issues of testing mediation with cross-sectional data and the limitations of our vigilance measure, we did not include these results in the paper, but they are available in the OSM for interested readers.

2. We reran all our analyses modeling emotional and behavioral jealousy for all participants, including those who did not provide valid open-ended responses to the jealousy recall question. The effect of relationship type on emotional jealousy became marginally significant, but all other results were similar to what we had found when including only participants with valid responses (see OSM Tables S2, S3, and S4).

3. We ran additional analyses in the sample of individuals in interracial relationships to examine whether there were differences in jealousy experiences among White versus non-White participants in interracial relationships involving one White partner. The results revealed no significant differences across all types of jealousy (see OSM Table S5).
